# Updates on aneurysmal subarachnoid hemorrhage: is there anything really new?

**DOI:** 10.1590/0004-282X-ANP-2022-S101

**Published:** 2022-08-12

**Authors:** Thire Baggio Machado Marazzi, Pedro Vitale Mendes

**Affiliations:** 1 Universidade de São Paulo, Departamento de Neurologia, São Paulo SP, Brazil.; 2 Universidade de São Paulo, Departamento de Emergências Clínicas, São Paulo SP, Brazil.

**Keywords:** Subarachnoid Hemorrhage, Aneurysm, Vasospasm, Intracranial, Hemorragia Subaracnóidea, Aneurisma, Vasoespasmo Intracraniano

## Abstract

**Background:**

Aneurysmal subarachnoid hemorrhage (aSAH) is a severe disease, with systemic involvement and complex diagnosis and treatment. Since the current guidelines were published by the AHA/ASA, Neurocritical Care Society and the European Stroke Organization in 2012-2013, there has been an evolution in the comprehension of SAH-associated brain injury and its multiple underlying mechanisms. As a result, several clinical and translational trials were developed or are underway.

**Objective:**

The aim of this article is to review some updates in the diagnosis and treatment of neurological complications of SAH.

**Methods:**

A review of PubMed (May, 2010 to February, 2022) was performed. Data was summarized.

**Results:**

Content of five meta-analyses, nine review articles and 23 new clinical trials, including pilots, were summarized.

**Conclusions:**

Advances in the comprehension of pathophysiology and improvements in critical care have been reflected in the reduction of mortality in SAH. However, despite the number of publications, the only treatments shown to be effective in adequate, well-controlled clinical trials are nimodipine and repair of the ruptured aneurysm. Thus, doubts about the optimal management of SAH still persist.

## RESUMO

**Antecedentes:**

Hemorragia subaracnóide aneurismática (HSAa) é uma doença grave, com envolvimento sistêmico, complexo diagnóstico e tratamento. Desde a publicação dos atuais protocolos de conduta pela AHA/ASA, Neurocritical Care Society e European Stroke Organization de 2012-2013, houve evolução na compreensão da lesão cerebral associada à HSA e seus múltiplos mecanismos subjacentes. Como resultado, muitos trabalhos clínicos e translacionais foram desenvolvidos ou estão em andamento.

**Objetivos:**

O objetivo deste artigo é revisar algumas das atualizações no diagnóstico e tratamento de complicações neurológicas de HSA.

**Métodos:**

Revisão de Pubmed (Maio de 2010 a Fevereiro de 2022) foi realizada. Dados foram sintetizados.

**Resultados:**

O conteúdo de 5 metanálises, 9 artigos de revisão e 23 novos estudos clínicos, incluindo pilotos, foram sumarizados.

**Conclusões:**

Avanços na compreensão da fisiopatologia e melhorias no cuidado crítico têm se refletido na redução da mortalidade em HSA. Entretanto, apesar do volume de publicações, os únicos tratamentos que se mostraram efetivos com testes clínicos bem controlados são o uso de nimodipino e o tratamento dos aneurisma rotos. Assim, dúvidas acerca do manejo ideal em HSA ainda persistem.

## GENERAL CONSIDERATIONS

Aneurysmatic Subarachnoid Hemorrhage (aSAH) is a complex disease and a relevant health problem. In terms of epidemiology, concepts should be highlighted: incidence varies greatly among countries. It is estimated at 2-16 per 100,000 per annum worldwide^1^, while Finland is the country with the highest incidence, with 22.7 per 100,000 per annum^2^. Furthermore, aSAH affects a relatively young population, with a peak of around 50 years-old^3^, causing higher mortality (about 50% at the time of aneurysmal rupture and with 30-day mortality up to 45%) and extensive long-term morbidity (a third of survivors require full care, and a third are not able to return to work)^4^. And lastly, it is estimated that the global aSAH case-fatality rate has decreased by 17% to 50% in the last 30 years as a result of improving diagnostic accuracy, surgical techniques, critical care support, cardiovascular risk prevention measures and adherence to guideline recommendations^5^. 

International guidelines are periodically updated with recommendations on screening, diagnosis, treatment and a comprehensive pathophysiological review. However, the last publication was by the AHA/ASA, Neurocritical Care Society, and the European Stroke Organization dated 2012-2013^3^ ^,^ ^6^ ^,^ ^7^. This article was developed by summarizing some recent studies (five meta-analyses, nine review articles and 23 clinical trials) and their results as to diagnosis and treatment of aSAH neurological complications.

## UP-TO-DATE IN DIAGNOSIS

In approximately 70% of patients with aSAH the clinical manifestation was a sudden headache. In order to help clinicians with diagnostic decisions in the emergency department the Ottawa SAH rules were developed. A cohort comprising 2131 patients with a headache peaking within an hour and no neurologic deficits was analyzed^8^. Ottawa SAH rules (Box 1) considered patients high-risk if one or more variables were present from some clinical and epidemiological criterias^8^. This tool, in practice, has reduced the total number of lumbar punctures^9^ in low-risk patients. The sensitivity was 100% (95% CI, 97.2%-100.0%) and specificity was 15.3% (95% CI, 13.8%-16.9%)^8^.

**Box 1.** The Ottawa SAH Rule*.

Inclusion: patients older than 15 y with new severe nontraumatic headache reaching maximum intensity within 1h.
Not for patients with new neurologic deficits, previous aneurysms, SAH, brain tumors, or history of recurrent headaches (≥3 episodes over the course of ≥6 mo).
Investigate if ≥1 high-risk variables present:
1. Age ≥40 y
2. Neck pain or stiffness
3. Witnessed loss of consciousness
4. Onset during exertion
5. Thunderclap headache (instantly peaking pain)
6. Limited neck flexion on examination

Adaptated of Perry JJ, Stiell IG, Sivilotti ML, et al. Clinical decision rules to rule out subarachnoid hemorrhage for acute headache. JAMA 2013; 310: 1248-55^8^.

* Ottawa SHA rules was a clinical decision tool.

## IMAGING

The imaging method recommended and most used for the diagnosis of SAH is the non contrast head Computed Tomography (CT). It is an easy-to-perform test with high sensitivity (93% to 100%) in the first six hours of symptoms^9^. The sensitivity of this method progressively reduces in the days following the ictus, when other modalities, such as cranial Magnetic Resonance Imaging (MRI), become more sensitive.

MRI is an imaging method that can be used from the hyperacute to the chronic phase^10^, requiring an adequate choice of sequence for analysis. More than two days after the ictus, the most used sequence is gradient recalled echo (GRE), reported in some studies with a sensitivity of 94% (95% for CT)^10^. Meanwhile, in subacute and chronic phases (4-15 days), the most sensitive sequences are susceptibility-weighted imaging (SWI) and fluid attenuated inversion recovery (FLAIR), sensitivities: 100% for FLAIR, 50% for CT, 30% for GRE^10^.

Generally, physicians prefer CT because of availability, lower costs and time and simpler MRI image acquisition in critically ill patients. However, MRI images provide a superior assessment of brain parenchyma and can be useful to predict unfavorable outcomes. De Marchis et al.^11^, even established that for every 10ml of DWI or FLAIR lesion volume, there was an outcome loss similar to 1 addition in Hunt Hess grade [OR 2.01 (95% (CI) 1.10-3.68; p=0.02)]. Other studies using functional outcomes by Rankin scale, cognitive test and Glasgow Outcome Scale have been described in a recent review^10^.

For the etiological diagnosis of SAH and programming an aneurysmal surgical approach, digital subtraction catheter angiography (DSA) with 3-dimensional reconstructions remains the gold standard. However, it remains an invasive and risky method. Alternatives are CT angiography (CTA), with a pooled sensitivity of 97% and specificity of 91%^12^, and magnetic resonance angiography (MRA). In meta-analysis^12^, MRA and CTA showed the same sensitivity as contrast-enhanced MR angiography (CEMRA) or time-of-flight MR angiography (TOF) technique. Nevertheless, some considerations must be made: MRA has higher rates of false-positives and false-negatives (especially lesions <3 mm and located at the skull base and middle cerebral artery)^12^ and MRA has low accuracy in aneurysm neck size determination^13^.New techniques have been developed to reduce coil artifacts and increase the already high sensitivity to residual aneurysm screening. One such technique was the sequence non-contrast enhanced zero echo time (zTE)^14^.

In recent years, MRI vessel wall assessment techniques have been studied to predict expansion and aneurysmal rupture, and to localize each high-risk in patients with multiple aneurysms^15^. Both qualitative and quantitative, automatic or semi-automatic methods of evaluating wall enhancement have been published, all with good predictive ability and good reproducibility^15^ ^,^ ^16^. There are still few studies showing a pathophysiological and radiological correlation associated with increased local vessel wall enhancement.

## UP-TO-DATES IN COMPREHENSIVE PATHOPHYSIOLOGY

SAH-associated brain injury (SAHBI) is still not completely understood despite medical advances made over the past three decades.

Previously, the SAHBI was didactically divided into early and delayed phases^9^. All studies focused on preventing and treating the most severe complications of each one. Management of unruptured aneurysms, reduction of risk factors, timing and surgical treatment techniques, treatment of rebleeding and hydrocephalus were the focus of early brain injury (EBI) trials. Meanwhile, in the delayed phase, prevention and treatment of vasospasm (VSP) were used in order to reduce delayed cerebral ischemia (DCI). 

As bench studies identified inflammatory mechanisms as precursors of DCI, some translational trials began to be developed. However, although the results demonstrated a reduction in large arteries VSP occurrence, there was no difference in functional outcome, e.g. clinical trials using the endothelin-1 (ET-1) receptor antagonist clazosentan^17^. These results motivated a shift in the focus of investigation from aSAH severe complications to the underlying mechanisms and the cascade triggered at the time of aneurysmal rupture and consequently downstream.

The current concept of pathophysiology of SAHBI is multiphasic, complex and multifactorial, with a cascade of events that are all interrelated and that permeate all stages of the disease^9^ ^,^ ^18^ ^,^ ^19^. Considered aSAH phases are a continuum in which all events contribute to outcome. 

Some supracited underlying mechanisms already studied were neuroinflammation, microthrombosis, cortical spreading depolarizations, disrupted integrity of the blood-brain barrier, microvascular dysfunction, sympathoadrenal activation and endothelial cell dysfunction. Many reviews on advances in each of these mechanisms and their promising fields of investigation have been published recently^18^ ^-^ ^20^.

## UP-TO-DATE IN NEUROLOGICAL MANAGEMENT

aSAH is a disease with severe neurological and systemic manifestations. Below are detailed some therapeutic and monitoring strategies for only neurological complications.

## REBLEEDING

At least ten randomized studies between 1982 and 2012 evaluated the use of oral or intravenous antifibrinolytic drugs (tranexamic acid, epsilon amino-caproic acid) for SAH early rebleeding prevention^21^. The results showed a reduced risk of rebleeding by about 35%, but no improvement in clinical outcomes. In addition, an increase in DCI was observed. Due to these two independent effects, current international guidelines differ in their recommendations about the use of antifibrinolytic drugs. To clarify this doubt, “Ultra-early Tranexamic Acid After SAH” (ULTRA) was developed and published in 2021^22^. Four hundred and eighty patients received ultra-early (at diagnosis) short-term tranexamic acid treatment (bolus 1g plus 1g each 8h, maximum doses 4g). No improvement in clinical outcome at six months was shown. Therefore, there is no evidence for current use.

## TIMING AND TREATMENT FOR ANEURYSM REPAIR

Guidelines suggest repairing the aneurysm “as early as feasible”(3), but it was still unclear whether ultra-early treatment (<24h) improves outcomes compared with early treatment (24-72h). Discordant results have been published in retrospective studies and the three largest^23^ ^-^ ^25^ were reviewed in meta-analysis^26^.Patients treated within 24 hours showed poor functional (OR 1.46 [0.47-2.9]) and mortality (OR 1.80 [0.88-3.67]) outcomes, when compared with those treated between 24 and 72 hours. This data should be critically evaluated: one (the largest sample) showed poor outcomes in treatment within 24 hours and all are retrospective, some non-randomized, most treated with coil. Thus, more studies are needed.

## EARLY BRAIN INJURY

Intravenous glibenclamide, a SUR1 inhibitor glyburide, has been shown to be safe and effective in reducing cerebral edema in patients with large cerebral infarct in pilot studies^27^. Some studies are underway with the use of the drug in patients with aSAH, including the Brazilian GASH trial^28^. Therefore, at the moment, there is no evidence to support its use

## DCI PREVENTION

### Strategies

Although prophylactic hypertension and hypervolemia are not recommended under current guidelines^3^ ^,^ ^6^ ^,^ ^7^, there are a few randomized controlled trials comparing the volume and pressure management strategies. Recently, a German group performed Randomized Controlled Trial (RCT)^29^ with 108 patients comparing goal-directed hemodynamic therapy (GDHT) versus standard therapy. Transpulmonary thermodilution monitoring was used to calculate global end-diastolic index, cardiac index and extravascular lung water index. According to an institutional goal protocol, fluids and vasoactive drugs could be used and titulated in accordance with clinical response or the occurrence of side effects. The results showed that GDHT reduced the rate of DCI (odds ratio: 0.324; 95% CI 0.11-0.86; p = 0.021), with a better functional outcome (GOS=5) three months after discharge, although it did not change the mortality rate when compared with the control group.

### Pharmacological therapies

Many pharmacological therapies have been tested for the prevention of EBI and DCI. However, most publication designs are retrospective studies or pilot trials. We summarize some of them and two RCTs in Table 1.

**Table1.** Clinical trials of delayed cerebral ischemia therapeutics.

Study	Study type	Agent	Biological Background	Result
STASH (Simvastatin in Aneurysmal Subarachnoid Hemorrhage)^31^	RCT	Simvastatin 40 mg/d orally for 21 days	Complex and multiple mechanism. Success phase II trials with others statins	No differences for long-term or short-term outcome
NEWTON2 (Study of EG-1962 Compared to Standard of Care Oral Nimodipine in Adults With Aneurysmal Subarachnoid Hemorrhage)^32^	RCT	Microparticle formulation of 600mg nimodipine. Application intratecal	Oral nimodipine improves clinical outcome, no reduction radiologic VSP	Trial stopped early due to high rate of vasospasm and DCI
Intraventricular Tissue Plasminogen Activator in Subarachnoid Hemorrhage Patients: A Prospective, Randomized, Placebo Controlled Pilot Trial^35^	Pilot trial Phase II	Dose 2 mg 12/12h intraventricular tissue plasminogen activator (TPA)	Amount of intracranial hemorrhage directly associated with worse clinical outcome.	TPA as a potent clot clearance accelerator. No clinical outcome assessment
Prospective, randomized, open-label phase II trial on concomitant intraventricular fibrinolysis and low-frequency rotation after severe subarachnoid hemorrhage^36^	Prospective randomized Phase II	5 mg of rt-PA was diluted in 2 mL of NaCl and given as an intraventricular bolus every 12 hours	Amount of intracranial hemorrhage directly associated with worse clinical outcome.	No reduction of delayed cerebral ischemia or poor functional outcome
Low-dose intravenous heparin infusion in patients with aneurysmal subarachnoid hemorrhage: a preliminary assessment^39^	Controlled retrospectively	Low-dose intravenous heparin infusion:8 U/kg/hr progressing over 36 hours to 10 U/kg/hr	Microthrombotic mechanisms in intracranial vasculature shown to be associated with DCI in bench study	Reduction in the occurrence of DCI and vasospasm in the intervention group. No increase in bleeding.

RCT: Randomized Clinical Trial; DCI: Delayed Cerebral Ischemia; VSP: vasospam.

*RCT findings*

Previously, the guidelines already included results from RCTs with the use of the magnesium sulfate (MASH II)(^30^) and endothelin-1 (ET-1) receptor antagonist clazosentan (CONSCIOUS 1 and 2)^17^ claiming no clinical benefit. After publication of the current guidelines, no new RCTs showed discordant results of MASH II over intravenous magnesium use. Recently, the use of clazosentanhas become a subject of study: the REACT trial is being developed with different clazosentan doses and it is proposed to identify the subgroups of patients who would benefit (ClinicalTrials.gov Identifier: NCT03585270) from prevention of neurologic worsening by DCI. 

Among the newly-published RCTs, two were more prominent: the use of oral simvastatin (STASH trial)^31^ and intrathecal use of nimodipine (NEWTON2 trial)^32^, both lacking favorable results in clinical outcome.

Therefore, unfortunately, no additional drug therapy has been suggested in high-quality studies.

*Therapies remain controversial*

The use of intraventricular fibrinolytic therapy had already been evaluated in meta-analyses in 2004^33^showing benefits in reducing DCI and morbidity. However, the quality of the nine studies included, with only one randomized, was considered low or moderate. Despite the limitations, the ASH treatment Japanese guideline^34^ incorporated the therapy into its recommendations. We found two subsequent published studies (Table 1), only one with a primary functional outcome^35^ ^,^ ^36^. In this study, the intraventricular fibrinolytic therapy had no benefits^36^.

*Emerging therapies*

Cilostazol, a selective phosphodiesterase-3 inhibitor with vasodilating and antiplatelet action, has been shown to be a promising and safe enteral drug.

A meta-analysis published in 2018^37^ evaluated the use of Cilostazol in four RCTs and a prospective cohort, in a total of 543 patients. The result was decreased risk of symptomatic vasospasm (0.31, 95% CI 0.20 to 0.48; P < 0.001), cerebral infarction (0.32, 95% CI 0.20 to 0.52; P < 0.001) and poor outcome (0.40, 95% CI 0.25 to 0.62; P < 0.001). No serious adverse effects were related with a dose of 100mg oral BID for 2 weeks. These studies however, included only those from the Japanese population. Most trials must be performed with another population.

Another promising therapy is continuous infusion unfractionated heparin, the use of which was associated with a reduction in rescue therapy necessity in severe vasospasm and DCI incidence, and improved cognitive outcomes^38^ ^,^ ^39^. In these, the dose used was started at 8 U/kg/h 12 hours after surgery, progressing in 36 hours to 10 U/kg/h (Maryland Protocol). The pathophysiological explanation is complex, as heparin has broad effects: antifibrinolytic and anti-inflammatory effects, reduction of free radicals, interaction with hemoglobin-free complex and activation endothelial.

An RCT is underway for large-scale evaluation of effects and safety: Randomizing Aneurysmal Subarachnoid Heparin Heparin Assay (ASTROH)^40^.

### Rescue therapies

In the treatment of established DCI, some rescue therapies are recommended. In this context however, no treatment was supported by a high-quality clinical trial and the impact of complications remains unmeasured. All recommendations were based on observational, retrospective, uncontrolled case series or institutional protocols.

Induction of arterial hypertension is the first treatment recommended by many guidelines in this scenario^3^ ^,^ ^6^ ^,^ ^7^. In 2018, the RCT^41^ compared functional outcome by Rankin scale among patients with and without induction of arterial hypertension three hours after onset of clinical symptoms. Hypertension was performed with norepinephrine or fluids, and was progressively increased until clinical improvement or MAP > 130 mmHg or SBP > 230, while the control maintained MAP around 80.

The study was paused with 41 participants due to slow recruitment and adverse effects. The adjusted risk ratio for poor outcome was 1.0 (95% confidence interval, 0.6-1.8) and the risk ratio for serious adverse events 2.1 (95% confidence interval, 0.9-5.0) was reported. 

Endovascular treatments with arterial balloon and intra-arterial vasodilator infusions, commonly used after hypertension induction due to favorable results in retrospective studies and case series, are not yet supported by RCT results. Venkatraman^42^ separated 55 studies using different doses and types (fasudil, nimodipine, nicardipine,papaverine verapamil) of intra-arterial vasodilators. The control group included patients without endovascular treatment or arterial balloon.Despite differences in outcome results with each vasodilator, all robustly reduced the severity of vasospasm but without neurological response. This study did not include milrinone as a vasodilator.

Milrinone is a selective inhibitor of the phosphodiesterase III isoenzyme with a vasodilatador and inotropic effect, which has been used as a rescue therapy after failure of induced hypertension in some specialized services in the world^4^ ^,^ ^43^, although it is not cited in current guidelines. Milrinone can be used as a continuous intravenous infusion (IV), intra-arterial (IA) bolus, or a combination of both (IVIA). Studies evaluating therapeutic modalities do not show differences in safety and outcome between intravenous or associated therapy^44^. In 2016, a meta-analysis found 24 studies using milrinone IV, IA, IVIA, all with low quality of evidence^45^. Unfortunately, the only RCT was discontinued in 2017 due to lack of suitable subjects^46^.

**Figure 1.**
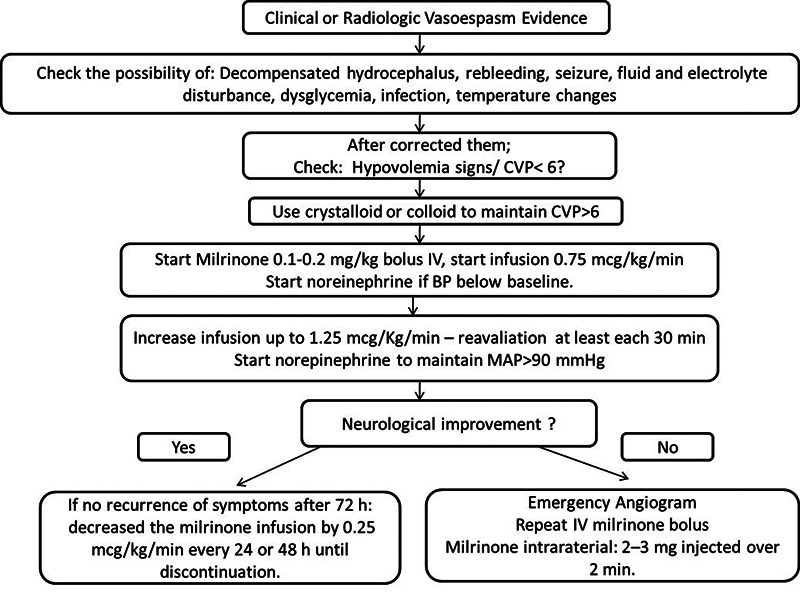
Milrinone use Montreal Protocol.

Specifically, the intravenous milrinone infusion protocol (initiation dose, continuous infusion dose, velocity of increment and withdrawal and treatment time) is based on service experiences, the most widespread being the Montreal Protocol (Figure 1) ^43^. There is still a lack of studies that evaluate the comparison of safety and benefit between intravenous infusion protocols from different institutions. 

Algorithm adaptation of milrinone using Montreal Protocol. CVP central venous pressure, BP blood pressure, MAP mean arterial pressure.

Recently, a retrospective study^47^ with 40 patients showed benefits without side effects with high doses of IV milrinone. In this study, 18 patients received boluses of up to 8mg IV with continuous infusion of up to 2.75 mcg/kg/min (maximum cumulative daily 230mg). 

Other inotropic therapies have been shown to be effective in reversing vasospasm. In a few comparative studies^48^ ^,^ ^49^, the benefit of using dobutamine outweighs that of milrinone in refractory patients.The risks and precautions are the same with both drugs: hypotension is the main complication and the use of a cardiac output monitor is the main additional care. 

For both drugs, high quality studies are needed.

## OTHER FREQUENT NEUROLOGICAL COMPLICATIONS

Despite the prevalence of seizures in SAH, no randomized clinical trials with new antiepileptic drugs for primary or secondary prophylaxis have been published.

In conclusion, advances in the comprehension of pathophysiology and improvements in critical care have been reflected in the reduction of mortality in SAH. However, despite the number of publications, the only treatments shown to be effective in adequate, well-controlled clinical trials are nimodipine and repair of the ruptured aneurysm. Thus, doubts about the optimal management of SAH still persist.
